# Prevalence and risk factors of postoperative delirium after spinal surgery: a meta-analysis

**DOI:** 10.1186/s13018-020-01651-4

**Published:** 2020-04-09

**Authors:** Hua Gao, Hui-Juan Ma, Ying-Jia Li, Ci Yin, Zheng Li

**Affiliations:** 1 Department of Outpatient, Lanzhou University Second Hospital, Lanzhou University Second Clinical Medical College, Lanzhou, 730030 China; 2 Operating Theater, Lanzhou University Second Hospital, Lanzhou University Second Clinical Medical College, Lanzhou, 730030 China; 3 Department of Thoracic Surgery, Lanzhou University Second Hospital, Lanzhou University Second Clinical Medical College, Lanzhou, 730030 China

**Keywords:** Delirium, Prevalence, Risk factor, Spinal surgery, Meta-analysis

## Abstract

**Objective:**

Postoperative delirium (POD) was common after spinal surgery, but the main findings in previous studies remained conflicting. This current meta-analysis was aimed at exploring the prevalence and risk factors of POD after spinal surgery.

**Methods:**

PubMed and Embase were searched from inception to June 2019. Studies which reported the prevalence and risk factors of POD after spinal surgery were included. STATA version 12.0 was employed to analyze the pooled data. Statistical heterogeneity across included studies was identified using the *I*^2^ statistics.

**Results:**

A total of 28 studies with 588,732 patients were included in the meta-analysis. The pooled prevalence of POD after spinal surgery was 0.85% (95%CI, 0.83–0.88%) with substantial heterogeneity (*I*^2^ = 97.3%). The central nervous system disorder (OR 4.73; 95%CI, 4.30–5.19) was a strong predictor for POD, whereas age (OR 1.16; 95%CI, 1.05–2.47; *I*^2^ = 99.2%) and blood loss (OR 1.10; 95%CI, 1.01–1.20; *I*^2^ = 93.3%) were weaker predictors. The funnel plot and statistical tests suggested that there existed potential publication bias, but the trim and fill method indicated that the pooled prevalence basically kept stable after adding two “missing” studies.

**Conclusions:**

The pooled POD after spinal surgery ranges from 0.83 to 0.88%. The central nervous system disorder, age, and blood loss were potential risk factors for POD.

## Introduction

Delirium, an acute state of confusion, is characterized as the distortion in consciousness and perception, decreased capacity of focusing one’s attention, deteriorated cognitive functions, and disturbed sleep-wake cycles [1, 2]. Postoperative delirium (POD) is a common complication after any major surgical procedure, which predominantly occurs in elderly [3, 4]. POD is associated with loss of independence, longer hospital stay, aggravated cognitive capacity, increased morbidity and mortality risk, and greater medical economic burden [5–7]. Unfortunately, the treatments for POD are full of challenges currently [3]. In general, identifying POD-associated risk factors is a potential useful way to understand the characteristics of POD, so it is essential to identify the potential perioperative risk factors which may help to establish effective strategies for prevention and treatment. In 2015, Shi et al. performed a meta-analysis to identify the POD-associated risk factors after spinal surgery [8]. However, the previous meta-analysis only included six studies, and thus, the reliability of its conclusion may be limited by the small sample size. Moreover, following the meta-analysis by Shi and coworkers, a large body of studies was performed to make a further exploration on the POD-associated risk factors after spinal surgery. Additionally, an accurate estimation of the POD incidence is also of much significance. On the one hand, in the intervention studies without a placebo group, a precise estimate of incidence is needed for comparison to determine whether the intervention could effectively prevent POD. The researchers could calculate the appropriate number of subjects needed for intervention studies based on the incidence [9]. On the other hand, an accurate estimate of incidence may help to identify some certain subgroups of patients, which would attract more attention of doctors to adjust interventions for the specific patient populations, and it meanwhile may guide researchers to establish the scientific inclusion of clinical trials, matching the interventional and control groups well in terms of POD-associated baseline risk variables. Nevertheless, currently, the reported prevalences of POD vary too widely following spinal surgery with inconsistency.

Therefore, in this meta-analysis, we combined the currently available studies to systematically examine the prevalence and risk factors of POD following spinal surgery.

## Methods

This meta-analysis was performed according to the guideline of the Meta-analysis of Observational Studies in Epidemiology checklist and the Cochrane Handbook [10]. Two reviewers separately performed selection criteria, data extraction, quality assessment, and statistical analysis, with inconsistence resolves by a third reviewer.

### Search strategy and selection criteria

A systematic literature search was performed in PubMed and Embase for studies published before June 10, 2019. The search terms included delirium, risk factor, spinal surgery, and their variants. Additionally, the reference lists of the eligible studies and relevant reviews were carefully screened to identify any potential inclusion. All eligible observational studies, which reported the incidence of delirium after spinal surgery or provided relevant information to calculate the incidence of POD, were assessed for inclusion in this meta-analysis. Studies enrolling fewer than 25 subjects, with overlapping patients, or without available data were excluded. When two or more studies included the overlapping populations, the one with the largest sample size and the longest duration was chosen for the current meta-analysis.

### Data extraction

Two co-authors independently extracted relevant data by using a pre-determined Excel sheet. The items of data extraction included the first author, year of publication, country, years of survey, type of operation, study design, mean age, sample size, risk factor, the number of delirium, and delirium assessment methods. The primary outcome is the prevalence of POD after spinal surgery. Additionally, the odds ratios (ORs) with corresponding 95% confidence intervals (CIs), which described the perioperative risk factors for POD after spinal surgery, were also extracted. Specially, we merely extracted individual risk factors which were assessed on multivariate or adjusted analysis in at least two studies.

### Quality assessment

The quality of eligible studies was evaluated using the Newcastle-Ottawa Scale (NOS) score [11, 12]. This score system is established specifically for assessing the quality of observational studies, in which scores are assigned for three dimensions, including selection criteria of participants, comparability, exposure, and outcome. A maximum score of NOS is up to 9, suggesting the highest quality.

### Data synthesis and analysis

Stata SE12.0 (Stata Corp., College Station, TX, USA) was used to estimate the pooled prevalence of POD after spinal surgery. According to the Cochrane Handbook (9.5.4), the random-effects estimate and its confidence interval address the question “what is the average effect?” while the fixed-effect estimate and its confidence interval address the question “what is the best estimate of the effect?” When the results of pooled analysis based on random-effects model and fixed-effect model are very different from each other owing to substantial heterogeneity across included studies, the fixed-effect estimate and its confidence interval, but not the random-effects estimate may more truthfully reflect the pooled prevalence of POD in spine surgery. Besides, sensitivity analyses were performed by excluding one study at each step to further assess the influence of individual included studies on the overall synthesis analyses. If individual risk factors of interest were reported in two or more studies, the pooled OR estimates with 95% CIs were calculated. Statistical heterogeneity across studies was evaluated using *I*^2^ statistic (*I*^2^ > 50% was regarded as substantial heterogeneity) [13, 14]. Subgroup analysis and meta-regression analysis for the primary outcome were used to detect the potential source of heterogeneity. The following categorical variables were analyzed in subgroup analyses: (1) region: Asia vs. Europe vs. North America; (2) year of the survey: before 2010 vs. after 2010 by the median splitting method; (3) sample size: ≤ 500 vs. > 500 by the median splitting method; (4) type of operation: oncological spine surgery vs. non-oncological spinal surgery; (5) mean age of patients: ≤ 60 vs. > 60; (6) study design: database analysis vs. non-database related observational study; and (7) preoperative disease status: with preoperative cerebrovascular disorders vs. without preoperative cerebrovascular disorders. Continuous variables including age, year of publication, and NOS score were analyzed by meta-regression analysis. Publication bias was assessed using Begg and Egger’s test and funnel plot [15, 16]. If there was significant publication bias, the “trim and fill method” was used to determine whether it obviously affected the robustness of the synthesis analysis [17]. *P* ≤ 0.05 was deemed to be statistically significant.

## Results

### Study selection and characteristics

**Fig. 1:**
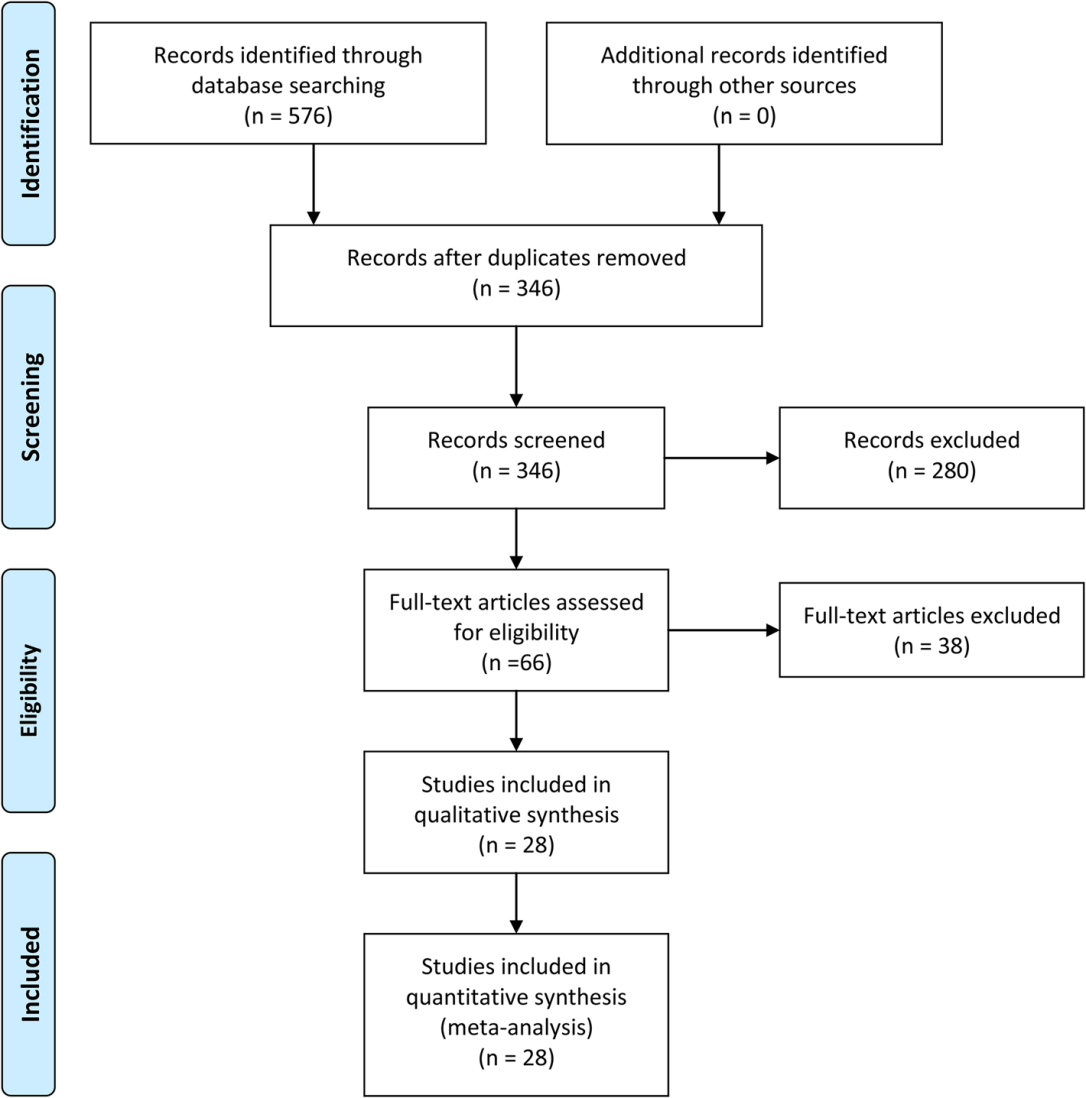
Flow diagram of the selection of reports for this meta-analysis

A total of 576 articles were identified initially. After removing duplicate records and irrelative titles and abstracts, the full texts of 66 articles were further screened for eligibility. Finally, a total of 28 studies with 588,732 patients were included in this meta-analysis [18–45]. The flow diagram for the study selection was presented in Fig. 1. The included studies were published from 2006 to 2019. Among all eligible studies, two were performed in Europe, nine in North America, and the others in Asia. The detailed characteristics of the included studies were shown in Table 1. The NOS score of all the included studies ranged from 6 to 9, suggesting the quality of included studies was relatively high for the current meta-analysis. The detailed NOS score of the included studies are shown in Table 2.

**Table 1:** Characteristics of included studies

Author,, year	Country	Year of survey	Type of operation	Age (mean, y)	Study design	Sample size	*n* (delirium)	Delirium assessment
Kawaguchi et al. 2006 [30]	Japan	2000–2002	Mixed spine surgery	59.2	Retrospective cohort study	341	13	CAM
Cho et al. 2007 [21]	Korea	NA	Degenerative lumbar scoliosis surgery	66.6	Retrospective cohort study	47	2	NA
Gao et al. 2008 [27]	China	(May–November) 2007	Mixed spine surgery	48.2	Cross-sectional	549	18	DOS
Ushida et al. 2009 [45]	Japan	2003–2007	Cervical surgery	69.8	Retrospective cohort study	122	26	DOS
Lee and Park 2010 [36]	Korea	2000–2007	Degenerative lumbar disease	73.5	Retrospective cohort study	217	11	CAM
Kelly et al. 2012 [31]	Canada	2009–2010	Degenerative spondylolisthesis surgery	66.08	Cross-sectional	92	5	NA
Li et al. 2012 [37]	China	2007–2011	Mixed spine surgery	75.3	Retrospective cohort study	1216	116	CAM
Bollen et al. 2013 [19]	The Netherlands	2001–2010	Spinal epidural metastases surgery	59	Cross-sectional	106	3	NA
Fineberg et al. 2013 [26]	The United States	2002–2009	Lumbar decompression and fusion surgery	55.1	Retrospective database study	578,457	4857	ICD-9-CM
Dea et al. 2014 [23]	The Netherlands	2009–2012	Oncological spine surgery	61.9	Cross-sectional	101	21	NA
Seo et al. 2014 [42]	Korea	2012–2013	Mixed spine surgery	70.1	Case-control study	70	17	DSM-5
Glennie et al. 2015 [28]	Canada	2009–2013	Thoracic and lumbar spine fracture surgery	44.3	Case-control study	276	38	NA
Brown et al. 2016 [20]	The United States	2012–2014	Mixed spine surgery	74	Case-control study	89	36	CAM-18, CAM-ICU19, and validated chart review
Radcliff et al. 2016 [41]	The United States	2010–2012	Cervical spine surgery	72.3	Retrospective database study	2792	157	NA
Elsamadicy et al. 2017 [24]	The United States	2005–2015	Spine deformity surgery	61.4	Retrospective cohort study	923	66	DSM-V criteria
Jiang et al. 2017 [29]	China	2010–2015	Mixed spine surgery	65.1	Cross-sectional	451	42	Clinical Dementia Rating and Global Deterioration Scale
Kobayashi et al. 2017 [35]	Japan	NA	Mixed spine surgery	Aged 80 years or older	Retrospective database analysis	262	15	NA
Soh et al. 2017 [43]	Korea	2014–2015	Mixed spine surgery	Aged 73 years or older	Prospective observational study	109	9	ICDSC and CAM-ICU
Adogwa et al. 2018 [18]	The United States	NA	Degenerative scoliosis surgery	Aged 65 years or older	Retrospective cohort study	82	22	CAM
Kim et al. 2018 [32]	Korea	2015–2016	Mixed spine surgery	71.7	Prospective cohort study	104	15	CAM
Kobayashi et al. 2018 [35]	Japan	2008–2013	Mixed spine surgery	91.3	Prospective database	35	11	NA
Morino et al. 2018 [38]	Japan	2012–2014	Mixed spine surgery	64.2	Retrospective cohort study	532	59	DSM-IV
Susano et al. 2018 [44]	The United States	2015–2017	Mixed spine surgery	73.6	Case-control study	716	127	NA
Cui et al. 2019 [22]	China	2016–2018	Mixed spine surgery	70.2	Case-control study	436	112	CAM
Elsamadicy et al. 2019 [25]	The United States	2010–2015	Mixed spine surgery	54.7	Retrospective cohort study	138	15	CAM
Kin et al. 2019 [33]	Japan	2014–2018	Surgery for cervical spondylotic myelopathy	69.6	Retrospective cohort study	67	10	CAM
Oe et al. 2019 [39]	Japan	2010–2017	Spinal deformity surgery	65.8	Retrospective cohort study	319	30	CAM
Pan et al. 2019 [40]	Korea	2015–2016	Lumbar spine surgery	71.4	Retrospective cohort study	83	12	CAM

*NA* no available, *DOS* delirium observation screening, *CAM* Confusion Assessment Method, *DSM* diagnostic and statistical manual of mental disorders

**Table 2:** The quality assessment according to the Newcastle Ottawa Scale of each study

Study	Selection	Comparability	Exposure	Total score
Kawaguchi et al. [30]	3	2	2	7
Cho et al. [21]	3	2	2	7
Gao et al. [27]	3	2	3	8
Ushida et al. [45]	2	2	3	7
Lee and Park [36]	3	2	2	7
Kelly et al. [31]	2	2	2	6
Li et al. [37]	3	2	2	7
Bollen et al. [19]	2	2	2	6
Fineberg et al. [26]	3	2	3	8
Dea et al. [23]	3	2	2	7
Seo et al. [42]	3	2	2	7
Glennie et al. [28]	3	2	3	8
Brown et al. [20]	3	2	3	8
Radcliff et al. [41]	3	2	3	8
Elsamadicy et al. [24]	4	2	3	9
Jiang et al. [29]	3	2	2	7
Kobayashi et al. [35]	3	2	3	8
Soh et al. [43]	3	2	3	8
Adogwa et al. [18]	3	2	2	7
Kim et al. [32]	3	2	3	8
Kobayashi et al. [35]	2	2	2	6
Morino et al. [38]	4	2	3	9
Susano et al. [44]	3	2	3	8
Cui et al. [22]	3	2	3	8
Elsamadicy et al. [25]	4	2	3	9
Kin et al. [33]	3	2	2	7
Oe et al. [39]	3	2	2	7
Pan et al. [40]	4	2	3	9

### Postoperative delirium after spine surgery

**Fig. 2:**
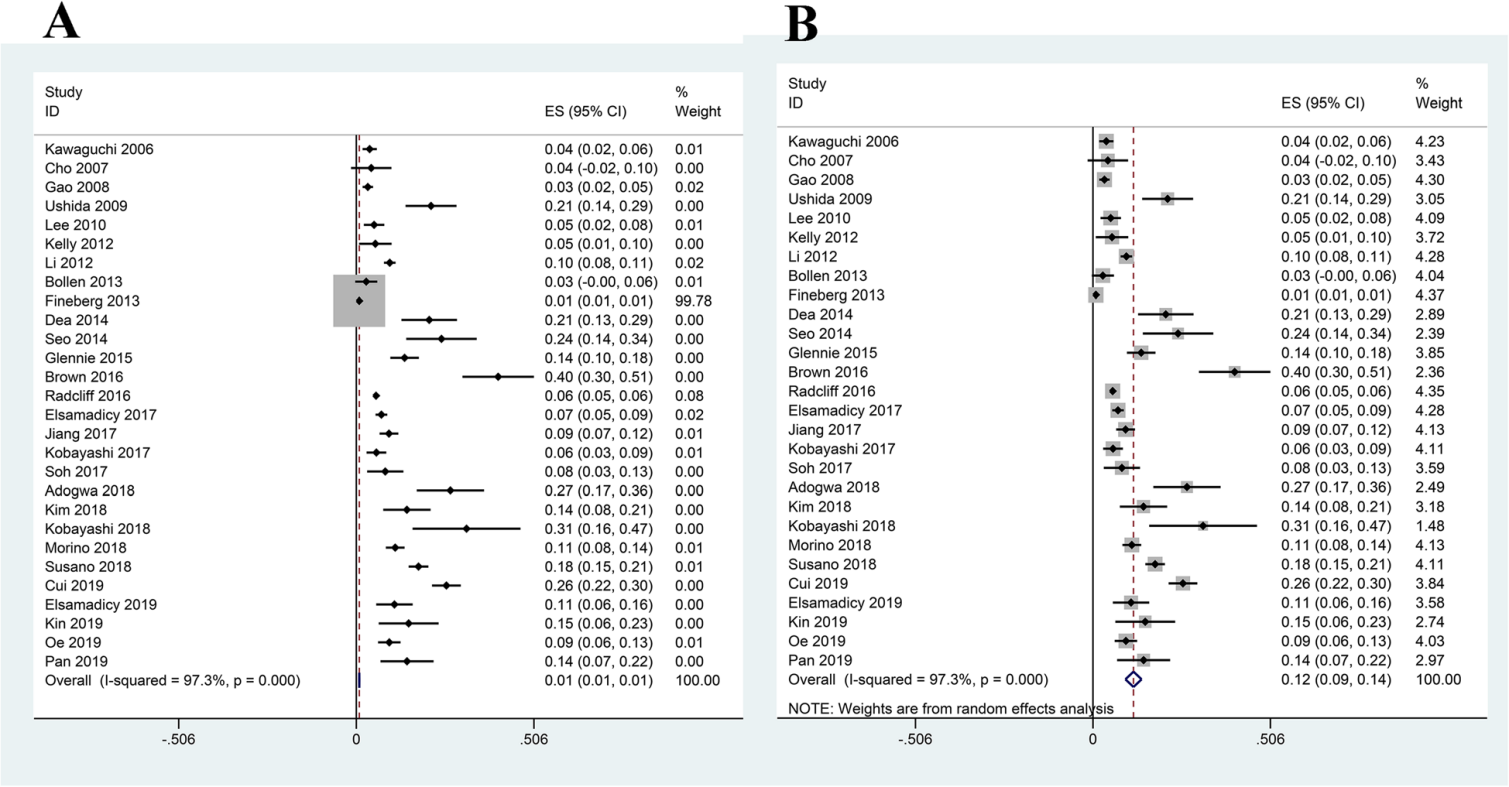
Forest plot for incidence of postoperative delirium after spinal surgery. **a** Fixed-effect model. **b** Random-effects model

**Fig. 3:**
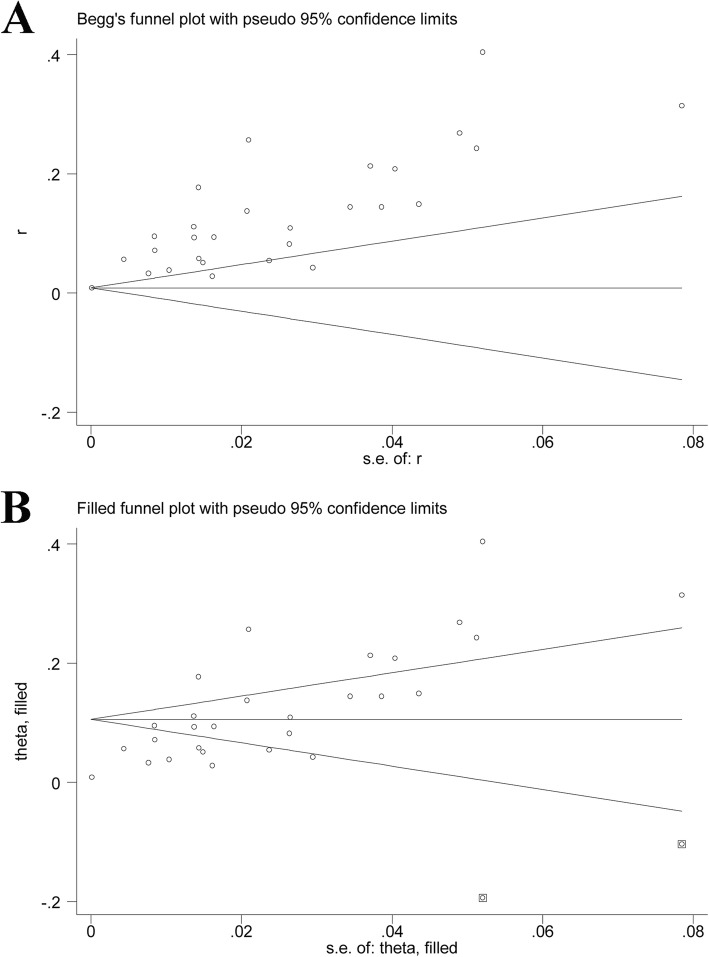
Funnel plot of postoperative delirium after spinal surgery (Egger’s test, *p* = 0.797 and Begg’s test, *p* = 0.008). **a** Adjusted funnel plot of postoperative delirium after spinal surgery after adding two “missing” studies from the “trim and fill” analysis (**b**)

All included studies reported POD after spinal surgery. The pooled prevalence of POD was 0.85% (95%CI, 0.0083–0.0088; *I*^2^ = 97.3%; Fig. 2a) using fixed-effect model, but the pooled prevalence based on random-effects model was 12% (95%CI, 0.09–0.14; *I*^2^ = 97.3%; Fig. 2b). According to the Cochrane Handbook, the fixed-effect estimate may more truthfully reflect the authentic pooled prevalence of POD in spine surgery when there existed a significant difference between the fixed-effect and random-effects estimates with substantial heterogeneity. Furthermore, we performed sensitivity analyses to explore the influence of individual included studies on the overall pooled effect. The results indicated that the pooled prevalence of POD basically remained stable except the pooled results (7.4%; 95%CI, 0.069–0.079) when excluding the study by Fineberg (Table 3). Subgroup analyses indicated that there were significant differences in the incidences of POD among Asia (7.7%), Europe (5.3%), and North America (0.8%), as well as between oncological spinal surgery (5.3%) and non-oncological spinal surgery (0.9%). In subgroup analyses stratified by year of survey, the incidence of POD after 2010 (4.1%) was higher than that before 2010 (0.9%). Similarly, in stratified analyses by mean age, we observed that the incidence of POD in patients older than 60 years (8.2%) was also higher than that in patients younger or equal to 60 years (0.9%). In subgroup analysis by study design, the incidence of POD in the subgroup of non-database related observational study (8.4%) was higher than that in the database analysis subgroup (0.8%). Subgroup analysis based on preoperative disease status showed that the incidence of POD in patients with preoperative cerebrovascular disorders (8.7%) was significantly higher than that in patients without preoperative cerebrovascular disorders (0.8%). When stratified by sample size, the incidence of POD in ≤ 500 group (8.8%) was higher than that in > 500 group (0.8%). The subgroup analyses were detailed in Table 4. Furthermore, the meta-regression analyses showed that publication time (*p* = 0.041), but not sample size (*p* = 0.183) and NOS score (*p* = 0.975), was significantly associated with higher POD after spine surgery. The funnel plot seemed to be asymmetric and statistical tests (Egger’s test, *p* = 0.797 and Begg’s test, *p* = 0.008; Fig. 3a) also suggested the significant evidence of publication bias. However, the pooled prevalence for POD (0.9%; 95%CI, 0.008–0.009) did not change significantly after adding two “missing” studies from the “trim and fill” analysis (Fig. 3b).

**Table 3:** Sensitivity analyses through removing individual studies each time

Study omitted	Estimate	LCI	UCI
Kawaguchi et al. [30]	0.00853848	0.00830359	0.00877338
Cho et al. [21]	0.00854187	0.00830699	0.00877675
Gao et al. [27]	0.0085364	0.00830149	0.00877131
Ushida et al. [45]	0.0085403	0.00830541	0.00877518
Lee and Park [36]	0.0085397	0.00830482	0.00877459
Kelly et al. [31]	0.00854126	0.00830637	0.00877614
Li et al. [37]	0.00852485	0.00828995	0.00875976
Bollen et al. [19]	0.00854134	0.00830645	0.00877623
Fineberg et al. [26]	0.07398792	0.06900876	0.07896708
Dea et al. [23]	0.00854068	0.0083058	0.00877556
Seo et al. [42]	0.00854115	0.00830627	0.00877603
Glennie et al. [28]	0.00853812	0.00830324	0.00877301
Brown et al. [20]	0.00854033	0.00830545	0.00877521
Radcliff et al. [41]	0.00850637	0.00827141	0.00874134
Elsamadicy et al. [24]	0.00852986	0.00829496	0.00876476
Jiang et al. [29]	0.00853595	0.00830106	0.00877083
Kobayashi et al. [35]	0.00853904	0.00830415	0.00877393
Soh et al. [43]	0.0085409	0.00830602	0.00877579
Adogwa et al. [18]	0.00854088	0.00830599	0.00877576
Kim et al. [32]	0.00854079	0.00830591	0.00877567
Kobayashi et al. [35]	0.00854172	0.00830684	0.0087766
Morino et al. [38]	0.0085345	0.00829961	0.00876939
Susano et al. [44]	0.00853053	0.00829565	0.00876542
Cui et al. [22]	0.00853429	0.0082994	0.00876917
Elsamadicy et al. [25]	0.00854038	0.0083055	0.00877527
Kin et al. [33]	0.00854137	0.00830649	0.00877625
Oe et al. [39]	0.00853784	0.00830295	0.00877272
Pan et al. [40]	0.00854112	0.00830624	0.008776

*LCI* low confidence interval, *UCI* upper confidence interval

**Table 4:** Subgroup analysis of delirium after spine surgery

Outcomes	Number of trials	Pooled prevalence with 95%CI	*I*^2^ (%)
Primary analysis	28	0.009 (0.008–0.009)	97.3
Subgroup analyses based on the region			
Asia	17	0.077 (0.069–0.084)	91.3
Europe	2	0.053 (0.024–0.082)	94.1
North America	9	0.008 (0.008–0.009)	98.3
Subgroup analyses based on the year of survey			
Before 2010	5	0.041 (0.030–0.052)	82.8
After 2010	23	0.009 (0.008–0.009)	97.7
Subgroup analyses based on the sample size			
> 500	7	0.008 (0.008–0.009)	98.8
≤ 500	21	0.088 (0.079–0.097)	90.9
Subgroup analyses based on the type of operation			
Oncological spine surgery	2	0.053 (0.024, 0.082)	94.1
Non-oncological spine surgery	26	0.009 (0.008–0.009)	97.4
Subgroup analyses based on the mean age of candidate patients			
> 60	22	0.082(0.077–0.088)	92.3
≤ 60	6	0.009(0.008–0.009)	93.2
Subgroup analyses based on the study design			
Database analysis	4	0.008(0.008–0.009)	98
Non-database related observational study	24	0.084(0.078–0.09)	92.4
Subgroup analyses based on the preoperative disease status			
Patients with not preoperative cerebrovascular disorders	28	0.008(0.008–0.009)	97.2
Patients with preoperative cerebrovascular disorders	3	0.087(0.079–0.093)	97.4

### Perioperative risk factors for postoperative delirium

A total of nine risk factors associated with POD after spine surgery were reported on multivariate or adjusted analysis in two or more included studies (Table 5). Of these risk factors, the central nervous system disorder (5 studies; OR 4.73; 95%CI, 4.30–5.19) was a strong predictor for postoperative delirium, whereas age (10 studies; OR 1.16; 95%CI, 1.05–2.47; *I*^2^ = 99.2%) and blood loss (four studies; OR 1.10; 95%CI, 1.01–1.20; *I*^2^ = 93.3%) were weaker predictors.

**Table 5:** Meta-analysis of risk factors for postoperative delirium in spine surgery

Outcomes	Number of trials	OR (95% CI)	*I*^2^ (%)
Age	10	1.61 (1.05–2.47)	99.2
Sex (men)	4	1.11 (0.36–3.46)	60.3
Hemoglobin < 100 g/L	2	0.61 (0.19–1.97)	76
Central nervous system disorder	5	4.73 (4.30–5.19)	0
Blood loss	5	1.10 (1.01–1.20)	93.3
Blood transfusion	2	2.57 (0.95–6.93)	0
Operative time	2	0.99 (0.97–1.01)	0
MMSE score	3	1.03 (0.62–1.69)	84.6
ASA score	2	4.25 (0.86–20.93)	92

*MMSE* Mini-Mental State Examination, *ASA* American Society of Anesthesiologists

## Discussion

The current study shows that the POD was a serious complication after spine surgery with pooled prevalence ranging from 0.83 to 0.88%. The central nervous system disorder was a strong predictor, whereas age and blood loss were the weaker predictors for POD after spine surgery.

In the present meta-analysis, a total of 28 studies were identified to evaluate the incidence of POD following spinal surgery. The overall pooled prevalence of POD was 0.85 % with substantial heterogeneity. We found that the incidence of POD in elder patients (> 60) was higher than that in younger patients (≤ 60), suggesting that older age might be a risk factor for POD after spinal surgery. In accordance with these, our data synthesis analysis revealed that older age was a significant risk factor for POD after spinal surgery (OR 1.16, 95%CI 1.05–2.47). In general, the elderly patients usually have poor general health status, more physical and psychological problems, and decreased functioning; all of which might contribute to the occurrence of POD after spinal surgery [46, 47]. Meta-regression analyses also found that publication time was significantly associated with higher POD prevalence. A possible interpretation was that the global aging population trend in these years may be an important contributor to the increased prevalence of POD accompanied with years [48]. Subgroup analysis also revealed that the pooled prevalence of POD in patients with preoperative cerebrovascular disorders was approximately two times higher than those without preoperative cerebrovascular disorders. Additionally, we found that the central nervous system disorder was identified as a strong predictor for POD after spine surgery as well. Actually, numerous previous studies have demonstrated that some cerebrovascular disorders including Alzheimer’s disease and dementia were associated with a high risk of delirium, which may help to explain our findings [49–52]. Our study also found that the male gender may be a significant risk factor for POD after spine surgery. A possible reason was that cerebrovascular disorders have a high prevalence among men versus women [53–55], which may lead to a high risk of POD after spine surgery. Furthermore, we identified that hemoglobin < 100 g/L, blood loss, and blood transfusion were potential predictors for POD. An important reason for this was that perioperative oxygen insufficiency of the central nervous system facilitated the development of POD after spine surgery. Other risk factors, such as operative time, MMSE score, and ASA score were possible risk factors for POD, although the pooled results were no statistical significant. Taken together, perioperative management focused on these aforementioned risk factors may reduce the risk of POD after spine surgery.

The current study also existed many limitations. Firstly, in the current study, the results of pooled analysis based on random-effects model and fixed-effect model are very different from each other owing to substantial heterogeneity across included studies. According to the Cochrane Handbook (9.5.4), the random-effects estimate and its confidence interval address the question “what is the average effect?” while the fixed-effect estimate and its confidence interval address the question “what is the best estimate of the effect?” Therefore, we just chose the fixed-effect estimate and its confidence interval, but not the random-effects estimate, which may more truthfully reflect the pooled prevalence of POD in spine surgery. The choice may be inappropriate, but can show the authentic pooled prevalence of POD in spine surgery.

Our study indicated that the overall pooled prevalence of POD was approximately 0.85% with substantial heterogeneity. Meta-regression analyses indicated that the year of publication was a significant contributor to statistical heterogeneity. Moreover, we performed subgroup analyses based on the year of survey and found that the incidence of POD after 2010 was lower than those before 2010. However, the statistical heterogeneity within subgroups was still substantial, so there may exist other significant sources of heterogeneity. Understandably, the statistical heterogeneity may not attribute to individual factors, such as publication time, but many clinical and methodological difference factors across included studies including demographic characteristics, type of operation, country, study design, and the definition or duration of POD. Secondly, most of the included patients in the current meta-analysis were from Steven J. Fienberg’s study which accounts for 98.25% of the total patients. Therefore, the results of the study may be potentially skewed in favor of statistics reported by Fineberg. Additionally, we also performed the sensitivity analyses by sequentially excluding single study and subgroup analyses to explore the robustness and creditability of our overall pooled effect. We found that there is a high possibility that our pooled result was skewed in favor of the statistics reported by Fineberg. Of course, we cannot totally exclude the bias risk since all the included studies were retrospective studies and unavoidable heterogeneity. Thus, further homogeneous and prospective studies should be warranted to elucidate the prevalence of POD following spine surgery. Thirdly, some risk factors were reported in limited included studies, but the pooled estimate based on limited studies may bias the authenticity. The pooled analysis based on two studies found that the ASA score was associated with more than four-time risk of POD after spine surgery, but with no statistical significance. Actually, many studies revealed ASA score was a significant predictor for POD [56–59]. Moreover, there are other factors like electrolyte imbalance and general condition of the patient before surgery which were not identified in the current meta-analysis, but may also be potential risk factors for POD. However, these factors were reported in the limited studies, so we did not include them in this meta-analysis for the pooled estimate, considering that the limited studies may bias the authenticity of our pooled analysis. Therefore, our results in this meta-analysis may be too conservative and should be interpreted cautiously. Meanwhile, many other potential risk factors for POD should be further assessed in future studies. Finally, the funnel plot and statistical tests suggested that the current meta-analysis may exist publication bias, regardless of the fact that we have performed a systematic literature search. However, the pooled prevalence for POD basically remained stable after adding two “missing” studies, which further supported the reliability of the pooled effect.

To sum up, our study indicated that the pooled POD after spinal surgery was approximately 0.85%. The central nervous system disorder, age, and blood loss were potential risk factors for POD. Further studies with more homogeneous clinical parameters should be warranted to illuminate the prevalence and risk factors of POD after spine surgery.

## Data Availability

All data are fully available without restriction.

## Abbreviations

CI: Confidence interval
OR: Odds ratio
POD: Postoperative delirium
